# Hospitalization patterns for adolescents with eating disorders during COVID-19

**DOI:** 10.1186/s40337-023-00867-z

**Published:** 2023-08-16

**Authors:** Leslie Schlapfer, Margaret A. Gettis, Valerie Dutreuil, Brooke Cherven

**Affiliations:** 1 Children’s Healthcare of Atlanta, 1001 Johnson Ferry Rd NE, Atlanta, GA USA; 2 Department of Pediatrics, Emory University School of Medicine, Atlanta, GA USA; 3 Aflac Cancer and Blood Disorders Center, Children’s Healthcare of Atlanta, Emory University School of Medicine, Atlanta, GA USA

**Keywords:** Anorexia nervosa, Eating disorders, Adolescents, COVID-19, Length of stay, Hospitalization, Boarding, Medicaid

## Abstract

**Purpose:**

The purpose of this study was to examine differences in clinical characteristics and hospital length of stay (LOS) for adolescents with eating disorders (EDs) requiring medical stabilization during the pre-COVID-19 and COVID-19 time periods.

**Methods:**

Medical record data were abstracted for patients with EDs hospitalized for medical stabilization between 1/1/2019–2/29/2020 (pre-COVID-19) and 3/1/2020–12/31/2021 (during COVID-19). Patient demographics, clinical characteristics and LOS were compared between COVID-19 eras. Patients were categorized as boarding if they remained hospitalized ≥ 1 day after medical stabilization. Multivariate negative binomial linear regression models were performed to determine incidence rate ratios (IRR) and 95% confidence intervals (95% CI) for factors related to increased LOS.

**Results:**

Of the 467 admissions during this study, 120 were pre-COVID-19 and 347 were during COVID-19. Monthly admissions for EDs were higher during COVID-19 versus pre-COVID-19 (15.8 vs. 8.6, *p* = 0.001). On multivariate analysis, factors associated with increased LOS included admission during COVID-19 (IRR 1.27, 95% CI 1.15–1.40), *p* = 0.001), boarding (IRR 1.77, 95% CI 1.63–1.93, *p* = 0.001), public insurance (IRR 1.12, 95% CI 1.01–1.23, *p* = 0.032), nasogastric tube usage (IRR 1.62, 95% CI 1.48–1.76, *p* = 0.001), heart rate < 40 beats per minute (IRR 1.21, 95% CI 1.11–1.33, *p* = 0.001) and abnormal electrocardiogram (IRR 1.25, 95% CI 1.14–1.37, *p* = 0.001).

**Conclusion:**

In addition to clinical factors, we found that admission during COVID-19, boarding, and public insurance were associated with increased LOS among patients with EDs. There is a need for greater availability of ED treatment centers to care for patients with EDs after medical stabilization.

## Abstract (plain language summary)

The COVID-19 pandemic has had a profound effect on the mental health of young people worldwide. This public health crisis has led to a significant increase in individuals seeking care for an eating disorder. In the United States, hospital admissions for adolescents with eating disorders requiring medical stabilization increased significantly during the COVID-19 pandemic. This study examines differences in the demographic and clinical characteristics of patients with an eating disorder hospitalized pre-COVID-19 and during the COVID-19 pandemic in one pediatric hospital in the United States. We found a significant increase in hospital admissions during the pandemic as well as longer hospital stays. Factors associated with prolonged hospitalizations include those patients with public insurance (Medicaid), nasogastric tube for delivery of nutrition, and low heart rate. We found no difference in demographic factors such as age, gender, and insurance as well as degree of malnutrition and medical severity between the two time periods. The shortage of appropriate eating disorder treatment options upon hospital discharge were especially challenging during the COVID-19 pandemic.

## Background

The COVID-19 pandemic, an unprecedented worldwide event, has developed into an ongoing public health issue. Over 95 million cases of COVID-19 have been diagnosed in the United States alone with over 1 million deaths [1]. Unvaccinated individuals and those with chronic health conditions remain especially vulnerable. Additionally, the pandemic has had profound implications for those suffering with eating disorders (EDs), complex biopsychosocial conditions with significant medical implications and in some instances, high mortality rates [2–7]. Treatment for these individuals requires a dual approach: medical intervention as well as psychiatric care. Unfortunately, the COVD-19 pandemic resulted in prolonged confinement with treatment interruptions, increased social isolation, and paused school and work routines. As a result, many individuals with EDs experienced a deterioration in their mental health with a concurrent increase in medical symptomatology [8].

For youth, the ages 11 through 21 years serve as the transition from childhood to adulthood. Expansive cognitive, physical, and psychosocial growth is characteristic of this time period. Successful navigation of these developmental milestones requires a stable home environment, adequate rest, nutrition, and healthcare along with opportunities for education, socialization, and physical activity [9, 10]. The COVID-19 pandemic imposed a number of preventative measures to combat the spread of the virus and to preserve individual health. For children and adolescents with EDs, these pandemic mitigation strategies were particularly difficult to traverse. School closures, cessation of outside gatherings and the move to online learning platforms affected these individuals’ perceptions of their body image, eating behaviors and physical activity [11, 12]. It has been reported by the Centers for Disease Control that the proportion of emergency department visits for eating disorders doubled among adolescent females during the period from January 2019 to January 2022 [13]. However, what is unclear is whether the increase in help seeking was due to a higher incidence of symptomatic people or greater recognition of an individual’s eating disorder because of more family time spent together in quarantine [14].

Studies concerning the effects of the COVID-19 pandemic on patients with pre-existing EDs are emerging. Continued examination of medical admissions during the pandemic are warranted as these studies could offer critical insights informing future healthcare decisions and treatment modalities. In addition, the boarding of patients after medical stabilization, has become an issue due to the lack of bed availability in ED treatment centers [15]. The aims of this study are to examine differences in clinical characteristics and hospital length of stay (LOS) for adolescents with EDs requiring medical stabilization during the pre-COVID-19 and COVID-19 pandemic time periods, prior to transfer to an ED treatment center or outpatient therapy.

## Methods

We conducted a retrospective chart review of all patients hospitalized for medical stabilization due to complications related to a known or suspected ED from 1/1/2019–2/29/2020 (pre-COVID-19 era) and 3/1/2020–12/31/2021 (during COVID-19 era) at one pediatric healthcare organization in the southeastern United States. The study was approved by the organization’s Institutional Review Board (STUDY# 00001360).

Adolescents admitted to the hospital for medical stabilization met criteria based on the Society for Adolescent Health and Medicine guidelines [2]. These patients were treated according to an institutional ED protocol which includes behavioral restrictions, medical interventions, standardized non-select menus, and nasogastric (NG) feedings for patients who are unable to complete meals or patients with severe malnutrition (mBMI < 70%) [16]. Patient charts were screened for eligibility for inclusion in the retrospective chart review. Inclusion criteria for the study were any patient with a primary diagnosis of anorexia nervosa (AN), including restricting type and binge-eating/purging type, bulimia nervosa (BN), or atypical anorexia nervosa (AAN) and placement on the ED protocol. Patients with other ED categories such as avoidant restrictive food intake disorder (ARFID) or other medical or psychiatric conditions were excluded due to differences in their treatment and discharge plans.

Data extracted from medical records were stored in a secure database approved by the healthcare organization. The following data were extracted from electronic medical records for patients admitted during the study period: sex, date of birth, insurance, LOS, admission heart rate (HR) (< 40 beats per minute or ≥ 40 beats per minute), presence of an abnormal electrocardiogram (EKG; e.g., prolonged QTc), and abnormal echocardiogram, pediatric intensive care unit (PICU) admission, and NG tube usage at any time during hospitalization, number of readmissions and number of days between readmissions, body mass index (BMI) on admission, and percent weight loss prior to admission, Malnutrition classification was determined based on the percent mBMI, BMI z-score, and the percentage of weight loss prior to admission [2]. For patients older than 20 years, the criteria from the Diagnostic and Statistical Manual of Mental Disorders 5th edition (DSM-5) was used to classify adult malnutrition [17].

Patient records were reviewed to determine the date that patients were deemed medically stable for transfer to an ED treatment center. The criteria for medical stability for transfer was dependent upon whether a patient would be transferring to residential treatment or an outpatient program. Patients discharging to an outpatient program need to demonstrate a higher threshold of medical stability, which includes stable vital signs of HR > 45 beats per minute, stable electrolytes and off phosphorus supplementation, stable blood pressures without symptoms of dizziness upon standing and off NG feeds. Residential treatment centers vary in their criteria for admission depending on whether they can accept patients requiring NG feeds or patients needing other medical interventions. Once a treatment center is selected and the patient is accepted, the patient is discharged from the hospital when notified by the accepting facility that a bed is available. Records were reviewed to determine the date the patient was deemed medically stable and number of days awaiting discharge was calculated; when this was ≥ 1 day, patients were categorized as *boarding*, meaning they were medically stable awaiting discharge. The definition of boarding is the practice of holding patients in emergency departments or pediatric inpatient units until an inpatient psychiatric bed becomes available [18].

### Statistical analysis

Descriptive statistics were computed for demographics and clinical characteristics of interest. We summarized continuous variables as means and standard deviations and/or medians and interquartile ranges. We calculated counts and percentages for categorical variables. Differences in continuous variables were tested using two-sample t-test (parametric) or Wilcoxon sum-rank test (nonparametric), respectively. Differences in categorical variables were tested using Chi-squared test and Fisher’s exact test, respectively.

We performed univariate and multivariate negative binomial linear regression models to assess clinical factors associated with the outcome of LOS days. We calculated incidence rate ratios unadjusted and adjusted for covariates and LS-means for categorical variables. All statistical analyses were conducted using SAS software V.9.4 and statistical significance was evaluated at the 0.05 threshold [19].

## Results

A total of 467 admissions for medical stabilization due to AN/BN/AAN were included in the study. There were 120 patient encounters pre-COVID-19 (25.7%) and 347 patient encounters during COVID-19 (74.3%). The average number of admissions per month was higher during the COVID-19 era than those in the pre-COVID-19 era in the sample (LS-Mean: 15.8 vs. 8.6, *p* = 0.001). Demographics and clinical characteristics during the study period are summarized in Table 1. There were no statistical differences pre-COVID-19 and during COVID-19 regarding age, sex, and insurance type. Most admissions were among females (89.7%), mean age at admission of was 15.2 years, and 78.4% had private insurance. Additionally, there were no significant differences between the two groups for the following clinical characteristics: abnormal echocardiogram (8.3% vs. 6%; *p* = 0.263), NG tube usage, (24% vs. 30%; *p* = 0.225), HR < 40beats per minute (39% vs. 43%; *p* = 0.437), PICU admission (13% vs. 12.1%; *p* = 0.725%), percent of weight loss prior to admission > 20%, (44% vs. 39%; *p* = 0.521), ED diagnosis (AN 81.7% vs. 81.6%, *p*-0.337), and malnutrition level, with most patients meeting criteria for severe malnutrition ( 49% vs. 42%; *p* = 0.485). The prevalence of an abnormal EKG was significantly higher in the pre- COVID-19 era compared to the during COVID-19 (71.7% vs. 55%, *p* = 0.002). There was no statistically significant difference in the proportion of patients who were classified as having more than one admission during the study period between the two groups (23% vs. 29%; *p* = 0.180).

**Table 1:** Demographic and clinical characteristics of patient encounters with comparison by pandemic period

	Entire Sample N = 467	Pre-COVID-19 Pandemic N = 120 (25.7%)	During COVID-19 Pandemic N = 347 (74.3%)	P value
Characteristic				
Sex				0.561^a^
Female	419 (89.7%)	106 (88.3%)	313 (90.2%)	
Male	48 (10.3%)	14 (11.7%)	34 (9.8%)	
Age at admission, in years				0.057^a^
Mean (SD)	15.2 (2.3)	14.8 (2.5)	15.3 (2.2)	
Median (IQR)	15.0 (14.0, 17.0)	15.0 (13.0, 16.5)	15.0 (14.0, 17.0)	
Insurance type				0.062^a^
Private	366 (78.4%)	91 (75.8%)	275 (79.3%)	
Public	88 (18.8%)	22 (18.3%)	66 (19.0%)	
No insurance	13 (2.8%)	7 (5.8%)	6 (1.7%)	
Abnormal EKG				**0.002** ^**a**^
Yes	278 (59.5%)	86 (71.7%)	192 (55.3%)	
No	189 (40.5%)	34 (28.3%)	155 (44.7%)	
Abnormal Echo				0.263^a^
Yes	29 (6.2%)	10 (8.3%)	19 (5.5%)	
No	438 (93.8%)	110 (91.7%)	328 (94.5%)	
NG Tube usage				0.225^a^
Yes	133 (28.5%)	29 (24.2%)	104 (30.0%)	
No	334 (71.5%)	91 (75.8%)	243 (70.0%)	
HR < 40				0.437^a^
Yes	197 (42.2%)	47 (39.2%)	150 (43.2%)	
No	270 (57.8%)	73 (60.8%)	197 (56.8%)	
Patient admitted to the PICU				0.725^a^
Yes	58 (12.4%)	16 (13.3%)	42 (12.1%)	
No	409 (87.6%)	104 (86.7%)	305 (88.0%)	
Length of stay in Days				**0.001** ^**b**^
Mean (SD)	11.0 (7.6)	7.8 (3.5)	12.1 (8.3)	
Median (IQR)	9.0 (7.0, 13.0)	7.0 (5.0, 10.0)	10.0 (7.0, 14.0)	
Readmission				0.180^a^
Yes	127 (27.2%)	27 (22.5%)	100 (28.8%)	
No	340 (72.8%)	93 (77.5%)	247 (71.2%)	
# Days between readmissions				**0.053** ^**a**^
< 30 days	20 (4.3%)	5 (4.2%)	15 (4.3%)	
30 or > days	51 (11.0%)	6 (5.0%)	45 (13.0%)	
No readmission	396 (84.8%)	109 (90.8%)	287 (82.7%)	
Patient Boarded				**0.001**
Yes	141 (30.2%)	13 (10.8%)	128 (36.9%)	
No	326 (69.8%)	107 (89.2%)	219 (63.1%)	
Number of days boarding				**0.032** ^**b**^
Mean (SD)	9.4 (9.3)	5.0 (2.5)	9.9 (9.6)	
Median (IQR)	7.0 (4.0, 11.0)	5.0 (2.0, 7.0)	7.0 (4.0, 12.0)	
% of Weight Loss				0.521^a^
> % 20	187 (40.0%)	53 (44.2%)	134 (38.6%)	
> % 15	95 (20.3%)	26 (21.7%)	69 (20.0%)	
> % 10	110 (23.6%)	23 (19.2%)	87 (25.1%)	
None	75 (16.1%)	18 (15.0%)	57 (16.2%)	
Level of Malnutrition				0.485^a^
Severe	205 (44.0%)	59 (49.2%)	146 (42.1%)	
Moderate	102 (21.8%)	26 (21.7%)	76 (22.0%)	
Mild	109 (23.3%)	25 (20.8%)	84 (24.2%)	
None	51 (11.0%)	10 (8.3%)	41 (11.8%)	
Eating disorder diagnosis				0.337^a^
Anorexia nervosa (AN)	381 (81.6%)	98 (81.7%)	283 (81.6%)	
Atypical anorexia nervosa (AAN)	74 (15.9%)	21 (17.5%)	53 (15.3%)	
Bulimia Nervosa (BN)	12 (2.6%)	1 (0.8%)	11 (3.2%)	

^*^Pre-COVID-19 19 Pandemic: Patients with an admission date 1/1/2019–2/29/2020 and COVID-19 Pandemic: Patients with an admission date of 3/1/2020–12/31/2021. For Level of Malnutrition, “None” is defined as patients who did not lose weight and did not have a percent weight loss. For % of weight loss, “None” is defined as patients who did not lose weight and did not have a percent weight loss

^†^All continuous summaries are presented: mean (SD), median (25th-75th percentiles). a = parametric *p*-values are calculated by two-sample t-Test; b = non-parametric p-values are calculated by Wilcoxon sum-rank test. Categorical summaries are presented as count and percentages. a = p-values are calculated as Chi-Squared test and b = p-values are calculated as Fisher’s exact test

^1^Anoxeria nervosa (AN) is defined as patient encounters who had F50.01 (restricting type) or F50.02 binge-eating/purging type patient diagnosis

^2^Atypical anorexia nervosa (AAN) is defined as patient encounters who had F50.8-Other specified feeding or eating disorder

^3^Bulimia nervosa (BN) is defined as patient encounters who had F50.2-Bulimia nervosa patient diagnosis

## Length of Stay

There was a significant increase in the mean LOS during COVID-19 compared to the pre COVID-19 era (12.1 ± 8.3 vs. 7.8 ± 3.5 days, *p* = 0.001, Table 2). A larger proportion of patients were boarded during COVID-19 (36.9%) compared to pre COVID-19 (10.8%, *p *= 0.0001). The number of days boarding was also significantly higher among admissions during COVID-19 (9.9 ± 9.6 days) compared to pre COVID-19 (5.0 ± 2.5 days, *p* = 0.032). We evaluated the association between length of stay in days with COVID-19 era, boarding status, age at admission, insurance, abnormal EKG, NG tube usage, and HR (< 40 vs. ≥40 beats per minute) in both univariate and multivariable models. In our univariate analysis, all independent variables except for age at admission, abnormal EKG, and ED diagnosis were significant predictors of LOS days (Table 2).

**Table 2:** Univariate and multivariate negative binomial linear regression for length of stay among adolescents with eating disorders

Characteristics	Univariable				Multivariate			
	Los (Days) LS-Mean (95% Cl)	Univ. IRR (95% Cl)	Overall P-value	Referent P-Value	Los (Days) LS-Mean (95% Cl)	Multivar. IRR (95% Cl)	Overall P-value	Referent P-value
COVID-19 (N = 467)			**0.001**				**0.001**	
Pre-COVID-19	7.83 (7.04, 8.69)	Ref.			10.13 (8.74, 11.75)	Ref.		
During COVID-19	12.09 (11.42, 12.81)	1.55 (1.37, 1.74)		**0.001**	12.89 (11.35, 14.64)	1.27 (1.15, 1.40)		**0.001**
Patient boarded (N = 467)			**0.001**				**0.001**	
No	8.44 (7.99, 8.92)	Ref.			8.58 (7.53, 9.79)	Ref.		
Yes	16.91 (15.71, 18.21)	2.00 (1.83, 2.20)		**0.001**	15.21 (13.22, 17.51)	1.77 (1.63, 1.93)		**0.001**
Age at admission, in years (N = 467)			0.128				0.480	
	--	0.98 (0.96, 1.01)		0.128	--	0.99 (0.98, 1.01)		0.480
Insurance (N = 467)			**0.011**				0.062	
Private	10.75 (10.13, 11.40)	Ref.			11.41 (10.25, 12.69)	Ref.		
No insurance	7.85 (5.65, 10.90)	0.73 (0.52, 1.02)		0.065	10.27 (7.83, 13.46)	0.90 (0.69, 1.17)		0.424
Public	12.50 (11.11, 14.06)	1.16 (1.02, 1.33)		**0.025**	12.75 (11.27, 14.42)	1.12 (1.01, 1.24)		**0.032**
Abnormal EKG (N = 467)			0.229				**0.001**	
No	10.57 (9.73, 11.48)	Ref.			10.23 (8.88, 11.77)	Ref.		
Yes	11.29 (10.55, 12.08)	1.07 (0.96, 1.19)		0.229	12.77 (11.17, 14.61)	1.25 (1.14, 1.37)		**0.001**
NG Tube usage (N = 467)			**0.001**				**0.001**	
No	9.12 (8.60, 9.66)	Ref.			8.99 (7.90, 10.23)	Ref.		
Yes	15.72 (14.45, 17.10)	1.72 (1.56, 1.91)		**0.001**	14.53 (12.60, 16.76)	1.62 (1.48, 1.76)		**0.001**
Heart rate < 40 (N = 467)			**0.050**				**0.001**	
No	10.50 (9.80, 11.26)	Ref.			10.37 (9.14, 11.78)	Ref.		
Yes	11.67 (10.78, 12.65)	1.11 (1.00, 1.26)		**0.050**	12.59 (10.87, 14.58)	1.21 (1.11, 1.33)		**0.001**
Eating disorder diagnosis (N = 467)			0.136				0.928	
Anorexia nervosa (AN)	11.27 (10.63, 11.93)	Ref.			11.56 (10.49, 12.75)	Ref.		
Atypical anorexia nervosa (AAN)	9.74 (8.52, 11.14)	0.86 (0.75, 1.00)		0.051	11.32 (9.90, 12.95)	0.98 (0.88, 1.09)		0.705
Bulimia Nervosa (BN)	10.25 (7.37, 14.25)	0.91 (0.65, 1.27)		0.579	11.40 (8.71, 14.92)	0.99 (0.76, 1.27)		0.912

**Fig. 1:**
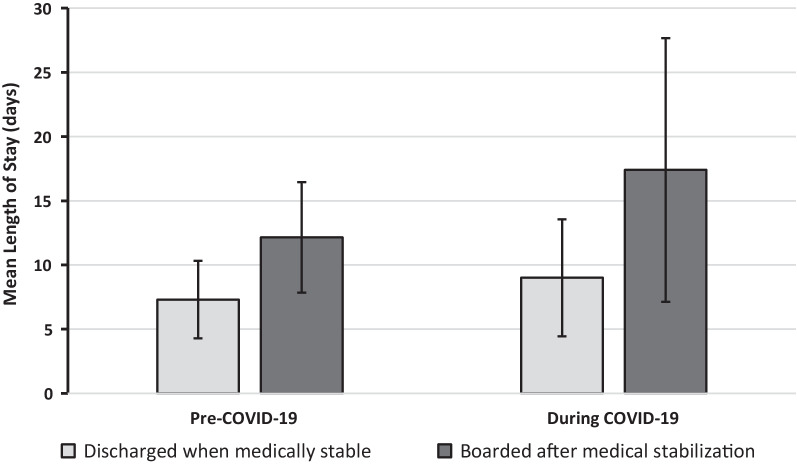
Length of stay pre- and during COVID-19 by discharge status. Pre-COVID-19: Patients with an admission date 1/1/2019–2/29/2020 and during COVID-19: Patients with an admission date of 3/1/2020–12/31/2021

On univariate analysis, patients who were admitted for eating disorders during the COVID-19 era had longer mean LOS days compared to the pre COVID-19 era (LS-Mean: 12.1 versus 7.8, *p* = 0.001), corresponding to a 55% longer admission (IRR: 1.55, 95% CI 1.37, 1.74, Table 2). Patients who were boarded, had 2.0 times longer admissions compared to patients who were able to be discharged when medically stable (IRR: 2.0, 95% CI 1.83–2.20; *p* = 0.001). The average LOS for patients who were boarded during the COVID-19 era was higher than those in the pre-COVID-19 era (17.4 ± 10.3 vs. 12.2 ± 4.3 days, Fig. 1). The incidence rate ratios for the remaining covariates are provided in Table 2.

In the multivariate negative binomial linear regression model, we examined patient characteristics associated with length of stay. After adjusting for other covariates, COVID-19 [IRR 1.27 (1.15–1.40), *p* = 0,001], boarding [IRR 1.77 (1.63–1.93), *p* = 0.001], public insurance [IRR 1.12 (1.01–1.23, *p* = 0.032)], NG tube usage [IRR 1.62 (1.48–1.76), *p* = 0.001] and HR < 40 beats per minute [IRR 1.21 (1.11–1.33), *p* = 0.001] remained significant. Patients with an abnormal EKG had 25% longer LOS compared to patients with no abnormal EKG [IRR 1.25 (1.14–1.37, *p* = 0.001)]. Age at admission was not a significant predictor of LOS days.

## Discussion

Our study findings show a nearly two-fold increase in hospital admissions for medical stabilization for adolescents with ED with a 55% longer length of stay during COVID-19 as compared to the pre-COVID-19 era. These findings are consistent with the literature worldwide on hospitalized youth with eating disorders that show an increase in hospitalization for ED during the COVID-19 pandemic [20, 21]. While admissions increased during the pandemic, the distribution of ED diagnoses remained similar, with > 80% of patients admitted with AN. We found a significant increase in the number patients who were boarded during the COVID-19 era, and the number of days awaiting discharge was nearly double that of pre-COVID-19 admissions. Multivariate analysis showed that public insurance (Medicaid), NG tube usage, and severe bradycardia (HR < 40) were associated with increased LOS.

The reasons for an increase in LOS and the proportion of ED patients who were boarded during COVID-19 are likely multifactorial, including barriers to accessing ED treatment centers. Hospitalizations for EDs increased during the pandemic without a corresponding increase in mental health providers or treatment facilities, resulting in a large gap in care for these patients [4, 20]. Options for residential ED treatment in the southeastern United States are limited, but even more so for patients with Medicaid insurance, as few ED residential treatment centers accept Medicaid insurance [8]. Often an out of state placement is the only placement option with availability, however, this presents an economic burden for families. While other institutions reported a decrease in the proportion of ED patients with Medicaid admitted during the pandemic, nearly 20% of patients at our institution pre- and during-COVID-19 had Medicaid, which substantially limits placement options [6, 8]. There are more treatment center options for patients with private insurance, including centers out of state, although available beds remain limited regardless of insurance status. In some cases, families may be able to discharge home to await ED treatment placement. However, patients discharging home need to demonstrate higher medical stability, including compliance with a nutritionally adequate diet without NG feeds; patients unable to meet these requirements remained hospitalized. Additionally, ED treatment centers during the pandemic required a negative SARS-CoV-2 test 24-hours prior to admission, which resulted in a 10-day delay in discharge for patients who tested positive [22].

Patients admitted for ED may also have significant psychiatric comorbidities including severe depression/anxiety and suicidality [23]. While the problem of children and adolescents boarding in pediatric hospitals while awaiting psychiatric placement (including placement at ED treatment centers) has been an issue for many years in the United States, the pandemic only exacerbated this crisis [18]. In addition to increased costs for hospital systems and families, these barriers to discharge result in a delay of care for specialized psychiatric treatment. Like many pediatric hospitals, this institution has an ED protocol, and the goal has been to provide medical stabilization and timely discharge to the next level of care. The psychiatry team provides evaluation, consultation, and treatment recommendations as well as supports patients and families during their hospitalization; however, patients are not provided with comprehensive ED psychotherapy. The prevailing thought has been that patients with severe eating disorders may not be fully able to engage in therapy until more medically stable. Given prolonged lengths of stay and the need to minimize exposure to other patients and staff as well as the COVID-19 visitor restrictions, many patients remained in their rooms with limited activities from one meal to the next. Protracted confinement without social interaction or activity may have exacerbated symptomology in these individuals [14].

Our study found no statistical difference in pre-COVID-19 and COVID-19 era regarding degree of malnutrition and PICU stay. These findings were unexpected and, given our high patient volumes, we hypothesized patients would be presenting to the hospital in a worsened nutritional state thus requiring PICU admission. In retrospect, we acknowledge that parents/caregivers may have been more aware of their child’s weight and eating behaviors as they were spending more time at home due to COVID-19 restrictions, thus seeking care earlier as noted in previous studies [5, 24]. We did find that patients with greater clinical needs (e.g., NG feeding and severe bradycardia) had longer LOS to reach medical stabilization.

While it is unknown whether the high volume of eating disorders admissions for children and adolescents will continue as the COVID-19 pandemic evolves, there is a critical need for greater availability and capacity at ED treatment centers (both residential and outpatient), particularly those that accept patients with Medicaid. The widely held belief that eating disorders affect mostly females of higher socioeconomic status has been largely debunked [25]. Despite this, very few treatment centers in the US accept patients with Medicaid leading to delays in treatment and limited access to specialized care for this vulnerable population. There is a need for nationwide efforts to increase the number of ED treatment centers that accept patients with Medicaid. In addition, pediatric hospital systems may need to augment their existing inpatient medical stabilization units with additional ED programming. Guidelines by the American Psychiatric Association and the Society for Adolescent Health and Medicine, call for comprehensive, individualized treatment plans managed by an experienced multidisciplinary team [26]. Possibilities could include pediatric institutions creating a day program to offer evidence-based ED support during admission while awaiting treatment center placement. However, once medical stabilization is achieved, patients must be able to progress to the next appropriate ED treatment setting in a timely fashion.

Our study has several limitations including it is a single institution study and retrospective in nature. In addition, there may be geographical differences in the availability of ED treatment centers which could impact the generalizability of these findings. Lastly, it is possible that the number of patients who were medically cleared but awaiting discharge is an underestimation, as we required a note in the chart stating the patient was medically stable to categorize the patient as boarding.

## Conclusion

Admissions for EDs increased during the COVID-19 pandemic, as did boarding of patients while waiting for placement in an ED treatment center. Longer LOS, particularly when due to boarding, results in unnecessary costs for the patient/family, increased cost for the medical institution, and a delay in comprehensive ED treatment for the patient. There is a need for greater availability of ED treatment centers to care for patients with EDs after medical stabilization.

## Data Availability

Data are available upon reasonable request.

## Abbreviations

AN: Anorexia nervosa
AAN: Atypical anorexia nervosa
ARFID: Avoidant restrictive food intake disorder
BMI: Body mass index
BN: Bulimia nervosa
DSM-5: Diagnostic and Statistical Manual of Mental Disorders 5th Edition
EDs: Eating disorders
EKG: Electrocardiogram
HR: Heart rate
LOS: Hospital length of stay
IRR: Incidence rate ratios
mBMI: Median body mass index
PICU: Pediatric intensive care unit
NG: Nasogastric
